# Systematic review of the effectiveness of self-initiated interventions to decrease pain and sensory disturbances associated with peripheral neuropathy

**DOI:** 10.1007/s11764-020-00861-3

**Published:** 2020-02-20

**Authors:** Theodora Ogle, Kimberly Alexander, Christine Miaskowski, Patsy Yates

**Affiliations:** 1 School of Nursing, Queensland University of Technology (QUT), Brisbane, Australia; 2 School of Nursing, University of California, San Francisco, CA USA

**Keywords:** Peripheral neuropathy, Pain, Self-management intervention, Patient reported outcomes, Quality of life, Symptoms

## Abstract

**Purpose:**

A small number of studies report that patients with peripheral neuropathy (PN) who engage in activities that promote a sense of personal well-being and provide physical, emotional, or spiritual comfort have a better quality of life and higher levels of adjustment to the changes generated by their illness and accompanying symptoms. This systematic review sought to evaluate the effectiveness of self-management activities that patients with PN initiate themselves to relieve PN symptoms and improve quality of life.

**Methods:**

Search terms were limited to include self-management activities initiated by patients (i.e., activities with no or minimal involvement from clinicians) that aim to provide relief of PN symptoms. Outcomes included in searches were pain, numbness, and tingling, associated with PN and quality of life.

**Results:**

The database searches identified 2979 records, of which 1620 were duplicates. A total of 1322 papers were excluded on the basis of screening the abstract. An additional 21 full text articles were excluded because they did not meet the eligibility criteria. A total of 16 papers were included in the review.

**Conclusion:**

This review identified that a number of self-management strategies that were initiated by patients, including heat, exercise, meditation, and transcutaneous electrical nerve stimulation (TENS) therapy, may reduce self-reported PN symptoms. As the available studies were of low quality, these strategies warrant further investigation with more homogeneous samples, using more rigorously designed trials and larger samples.

**Implications for Cancer Survivors:**

Patients experiencing PN may find a range of self-initiated strategies beneficial in reducing PN symptoms and improving quality of life. However, because of the low quality of the available studies, clinicians need to monitor patients’ responses to determine the effectiveness of these interventions as adjuncts to clinician-initiated interventions.

## Introduction

Peripheral neuropathy (PN) is a term that is used to describe a range of neuropathic symptoms that arise from disruptions in the structure and function of peripheral sensory, motor, and autonomic neurons [1, 2]. Studies from the USA and Europe report that PN occurs in 2.4% of the general population and increases to 8% in those over 55 years of age [3]. PN occurs in a number of chronic medical conditions (e.g., diabetes) or as a result of exposure to neurotoxic drugs [4–7]. In terms of specific medical conditions, approximately 50% of patients with human immunodeficiency virus (HIV) [8], 26% of patients with diabetes [9], and 30 to 40% of oncology patients who receive neurotoxic chemotherapy [10] report PN symptoms.

Typically, patients with PN report tingling and numbness, as well as burning or shooting pains during and following the course of the disease and treatment [7, 11–14]. Regardless of its etiology, effective pharmacological treatments for PN are not available [15, 16]. Pain associated with PN is not effectively treated with opioid or adjuvant analgesics [11, 13, 17, 18], and some of these treatments have side effects that outweigh their potential benefits [9]. While reductions in PN-related pain were found with anti-depressants (venlafaxine [19], duloxetine [20]), results vary considerably among patients. Anti-convulsants (e.g., pregabalin, gabapentin) show similar variations in efficacy [21]. To manage the symptoms of PN, which often persist for several months to years, patients use a range of nonpharmacological treatments [22–25]. While acupuncture [26], whole-body vibration [27], physical therapy [28], reflexology massage [29], and light therapy [30] have been evaluated as treatments for PN, responses are highly variable.

Peripheral neuropathy can be a chronic condition for many patients. While pharmacological and nonpharmacological treatments for PN have variable success [9, 31, 32], the potential benefits of self-management strategies to decrease chronic PN symptoms and improve function and quality of life are not well described [33–40]. In particular, the potential benefits of nonpharmacological interventions that incorporate patient self-management have received limited attention. That is, self-management refers to specific, targeted, intentional actions that individuals engage in to manage the symptoms and outcomes associated with specific diseases or to prevent the reoccurrence of symptoms or functional deficits [34, 37, 40]. In the context of PN, self-management could include steps that patients take to reduce PN-related symptoms, including pain, numbness, or tingling [22–24, 41].

The value of self-management strategies for patients with chronic conditions in general is well documented. Findings suggest that patients with chronic conditions who prioritize and engage in self-management activities that promote a sense of personal well-being and provide physical, emotional, or spiritual comfort report better quality of life and higher levels of personal adjustment to the changes generated by their illness and accompanying symptoms [36, 37, 39].

Self-management activities to relieve disease or treatment-related symptoms can be classified as either supported self-management (i.e., those that are initiated and require support and education by a clinician) or self-initiated strategies (i.e., those that patients initiate independently). Self-initiated actions are described as those that arise from an internal decision to take some form of action, usually in response to an internal stimulus [42]. Self-initiated strategies for PN symptoms include massage, heat therapy, cold therapy, magnet therapy, exercise, walking, rest, meditation, and transcutaneous electrical nerve stimulation (TENS) [22–25]. Patients who engage in these strategies aim to reduce the severity of their symptoms (e.g., pain, numbness, tingling, allodynia) and increase overall comfort and well-being [22–25]. Heat therapy, massage, and TENS are examples of self-initiated self-management measures that are used by patients who experience pain, including PN-related pain, because of their relatively low cost, ease of access, and perceived efficacy of such interventions [23–25, 43–47].

The purpose of this review is to evaluate the perceived effectiveness of self-initiated, self-management strategies in reducing PN symptoms. For the purpose of this review, a “self-initiated self-management strategy” is defined as an activity that patients can initiate independently and maintain on their own, with no required input from clinicians, to provide comfort or relief from the symptoms of PN and improve quality of life. Self-initiated strategies such as heat/cold therapy, massage, meditation, exercise, and TENS therapy are activities that people experiencing PN symptoms can attempt regardless of their financial or geographic status or access to health services. This review thus complements other reviews which have examined the efficacy of medication [9, 48, 49], and supplements and integrated therapies typically prescribed or administered by clinicians [41, 50–52]. Other reviews have examined self-initiated strategies including exercise [53–55] and TENS [56] on their own. As such, this review adds to existing literature by focusing on those activities which patients can self-initiate in relation to managing PN symptoms, and synthesizing findings from the various activities which are classified within this definition.

## Methods

This systematic review summarizes the results of 16 quantitative studies. Methods suggested in the Joanna Briggs Institute (JBI) manual for systematic reviews directed the process of conducting and reporting this review [57, 58]. Previous research has identified a range of self-initiated self-management activities that people experiencing PN symptoms report attempting to alleviate symptoms and increase well-being [22–25]. The search strategy for this review has been informed by that research and focuses on a more restricted range of activities that may be considered self-initiated self-management activities in that patients can initiate and maintain the activities with no required input from clinicians.

### Search strategy

Databases searched included Cumulative Index of Nursing and Allied Health Literature (CINAHL), Cochrane Library, Embase, Joanna Briggs Institute, MEDLINE, and Scopus. Where search processes allowed, PN was searched within the article title, with all other search terms included in separate searches under title/abstract across articles. Studies were included if patients had PN with pain, discomfort, numbness, or other sensory disturbance. Patients with PN from any disease or treatment-related etiology were included in this review. The review considered studies that included the following self-reported outcome measures: pain, numbness, tingling, and/or allodynia associated with PN and quality of life.

### Inclusion and exclusion criteria

Studies were included if they met the following inclusion criteria:

- Published in English between 1946 and April 2019
- Evaluated one or more of the following self-initiated self-management behaviors that were identified in the available literature for PN: meditation, cold therapy, heat therapy, magnet therapy, massage, exercise, walking, rest, or TENS
- Included people with symptomatic PN from diabetes (DPN), HIV, chemotherapy-induced peripheral neuropathy (CIPN), chronic inflammatory demyelinating neuropathy (CIDN), and autoimmune disorders
- Used an experimental study design (RCT, non-RCT, and quasi-experimental studies)
- Included adult patients
- Evaluated any of the following self-reported outcomes associated with the self-management intervention for PN: pain, numbness, tingling, and/or allodynia associated with PN and quality of life

Studies were excluded if the self-management strategies evaluated:

- Could only be performed under the direction of clinicians or other health professionals (i.e., were not self-initiated)
- Involved the ingestion of pharmacological, herbal, or other substances as these would require clinician input to assess for interaction with the medications used in the treatment of their medical condition
- Occurred with patients who had PN arising from trauma or other conditions not listed in the inclusion criteria

### Search terms

Search terms included PN AND meditation OR hypnosis OR imagery; PN AND ice OR cold; PN AND magnet; PN AND massage; PN AND exercise; PN AND walking; PN AND rest; PN AND TENS; PN AND heat OR hot; PN AND bath*. Medical subject headings (MeSH) were used when available. The reference lists of all identified papers were then searched for additional studies. Final searches were performed on 20 April 2019.

### Screening and data extraction

Titles were screened for inclusion according to the criteria specified above. Abstracts of potentially eligible citations were screened by two reviewers independently, with discrepancies discussed until agreement was reached. References within systematic reviews were screened for eligibility; however, systematic reviews were excluded. Following the removal of articles which did not meet the initial/abstract screening, the full text of the remaining articles was obtained and screened by two reviewers independently. For those studies that were deemed eligible for inclusion, data were extracted including year of publication, study purpose and design, sample characteristics, intervention and control applied, outcome measures, key findings, and strengths and limitations (Tables 1, 2, 3, 4, and 5).

**Table 1:** Demographic characteristics of participants in included studies

Type of intervention	First author (year)	Design	Cause of PN	Grade of PN	No. of subjects				Age (years) (*M*, SD)			Female
					IG		CG		IG	CG		
Hot bath/massage	Park and Park (2015) [59]	Experimental	CIPN	Grade II = 58.3% Grade III = 41.7%	24		24		57.08, 11.52	60.79, 8.97		37.5
Meditation	Clark et al. (2012) [60]	RCT	CIPN	Not specified	A 7	B 7	C 5	D 7	59.04, 8.56			88.5
	Nathan et al. 2017 [63]	RCT	CIPN	*M* = 5.1 (SD *=* 1.3) on BPI	30		32		59.7, 9.1	59.8, 8.7		56
	Teixeira (2010) [62]	RCT	CIPN	Not specified	10		10		74.6, 10.8			75
Exercise	Ahn and Song (2012) [﻿^67^]	Experimental	DPN	Not specified	20		19		66.05, 6.42	62.73, 7.53		48.7
	Dixit et al. (2014) [66]	RCT	DPN	Minimum score of 7 on MDNS	29		37		54.40, 1.24	59.45, 1.16		43.6
	Kluding et al. (2012) [64]	Experimental	DPN	Not specified	17				58.4, 5.98			53
	McCrary et al. (2019) [68]	Experimental	CIPN	≥ 2 on CTCAE v.4.03	29				61.6 (*M*), 32–79 (range)			72.4
	Ruhland and Shields (1997) [65]	Experimental	CIDN	Not specified	14		14		63.6, 10.5		52.9, 16.2	32
	Yoo et al. (2015) [61]	Experimental	DPN	Not specified	14				57, 5.11			64.3
TENS	Forst et al. (2004) [70]	RCT	DPN	≥ 4 and ≤ 16 on NTSS-6	12		7		57.6, 11.5		59.4, 8.6	47
	Gewandter et al. (2018) [72]	Experimental	CIPN	≥ 4 on NRS 1–10	22				Mdn 56, IQR 53–63			59
	Kumar & Marshall (1997) [69]	RCT	DPN	Not specified	18		13		53, 4		59, 3	61
	Reichstein et al. (2005) [74]	RCT	DPN	TSS: HF *M* = 7.0, SD = 3.6 TENS *M* = 6.6, SD = 3.2	HF 20		TENS 21		HF 64.2, 12.7		TENS 57.8, 12.5	43.2
	Serry et al. (2015) [71]	RCT	DPN	Not specified	A 20	B 20	C 20		A 51.6, 4.75	B 51.7, 4.44	C 51.7, 4.44	53
	Tonezzer et al. (2017) [73]	RCT	CIPN	Grade I or II on CTCAE	11		13		52.7, 9.0		46.3, 13.7	20.8

*IG* = intervention group; *CG* = control group; *M* = mean; *SD* = standard deviation; *RCT* = randomized controlled trial; *PN* = peripheral neuropathy; *CIPN* = chemotherapy-induced peripheral neuropathy; *DPN* = diabetic peripheral neuropathy; *CIDN* = chronic inflammatory demyelinating neuropathy; *BPI* = Brief Pain Inventory; *MDNS* = Michigan Diabetic Neuropathy Score; *NTSS-6* = New Total Symptom Score; *NRS* = Numerical Rating Scale; *TSS* = Total Symptom Score; *Mdn* = median; *IQR* = interquartile range; *HF* = high-frequency muscle stimulation; *TENS* = transcutaneous electrical nerve stimulation; *CTCAE* = Common Terminology Criteria for Adverse Events

**Table 2:** Self-reported symptom outcomes: summary of measures and results

First author (year)	Purpose and intervention	Outcome measures	Main findings
Ahn and Song (2012)[67]	*Purpose*: Determine the effects of tai chi exercise on glucose control, neuropathy scores, balance, and quality of life in patients with diabetic PN. Intervention: 1 h of tai chi per session, twice a week for 12 weeks. Control: Usual care	Total Symptom Score (TSS) questionnaire	Differences in pre- and post-test mean TSS indicated significant symptom improvement in the intervention group (mean change from *M* = 1.13, SD = 1.95 to *M* = 0.91, SD = 1.87 vs *M* = 1.19, SD = 1.98 to *M* = 2.83, SD = 3.29 for the controls, *p* = 0.042).
Reichstein et al. (2005)[74]	*Purpose*: Compare the effects of high-frequency muscle stimulation (HF) with TENS therapy in patients with diabetic PN. HF group received HF for 30 min for 3 consecutive days. TENS group received TENS therapy for 30 min for 3 consecutive days.	Total Symptom Score (TSS) questionnaire	Differences in pre- and post-test mean TSS indicated significant symptom improvement in both groups: HF group mean reduced from *M* = 7.0, SD = 3.6 to *M* = 4.6, SD = 3.4, (*p* < 0.005); TENS group *M* = 6.6, SD *=* 3.2 to *M* = 5.4, SD = 3.8, (*p* < 0.05).
Dixit et al. (2014)[66]	*Purpose*: Evaluate the effect of an 8-week moderate-intensity aerobic (heart-rate reserve 40–60%) exercise program on neuropathy-related quality of life in people with diabetic PN. Intervention: Exercise training in the range of 40–60% of heart-rate reserve (HRR) within a rating of perceived exertion (RPE) (scale ranging from 6 to 20). Delivered 5–6 days of the week for 8 weeks, accumulating a minimum of 150 min/week to a maximum of 360 min/week. Control: Standard medical care, education for foot care and diet	Michigan Diabetic Neuropathy Score (MDNS)	Mean MDNS score significantly reduced across sensory, motor, and reflex subsets for the intervention group between baseline and the 8th week (*M* = 12.57, SD = 1.74, 95% CIs [13.11–12.03] reduced to *M* = 7.03, SD = 1.86, 95% CIs [7.61–6.45], *p* < 0.001), in comparison to an increase in the control group (*M* = 13.55, SD = 1.75, 95% CIs [14.05–13.05], increased to *M* = 14.57, SD = 1.50, 95% CIs [15–14.09], *p* < 0.001), indicating an improvement in PN symptoms in the intervention group.
Kluding et al. (2012)[64]	*Purpose*: Examine the feasibility of a supervised, moderately intense aerobic and resistance exercise program in people with diagnosed diabetic PN. Intervention: A 10-week exercise program with both aerobic and strengthening elements, 3 to 4 times per week. Control: None	Michigan Neuropathy Screening Instrument (MNSI) symptom questionnaire	Overall mean MNSI symptom score decreased significantly (*M* = 5.24, SD = 1.4 pre-intervention to *M* = 4.00, SD = 2.00 post-intervention, *p* = 0.01), indicating an improvement in PN symptoms.
Gewandter et al. (2018)[72]	*Purpose*: Inform a future randomized phase 2 study and determine if TENS has the potential to improve CIPN. Intervention: Wireless TENS therapy for at least 1 h twice per day for a 6-week period. Control: None	European Organization for Research and Treatment of Cancer-CIPN20 (EORTC-CIPN20)	EORTC-CIPN20 median baseline scores reduced from 39.5 (IQR 31–47.3) to Mdn 34.5 (IQR 28.8–41.8, *p* = 0.004) post-TENS intervention, indicating and improvement in PN symptoms. Median SF-MPQ-2 scores reduced from baseline Mdn 60.5 (IQR 21.8–88.5) to a post-intervention Mdn 29 (IQR 8.8–63.8) (*p* < 0.001), indicating an improvement in self-reported pain of 52%.
McCrary et al. (2019)[68]	*Purpose*: Evaluate the impact of a multimodal exercise intervention on CIPN symptoms, functional deficits, and neurophysiologic parameters. Intervention: An 8-week exercise intervention with resistance, balance, and cardio elements, 3 times per week. Control: An 8-week pre-intervention control period	CIPN symptom severity (European Organization for Research and Treatment of Cancer [EORTC] CIPN 20); Overall Disability (CIPN Rasch Built overall disability score [CIPN-R-ODS])	Significant reduction in CIPN symptoms (CIPN-20 score) (*M* = 25.4, SE = 3.0 pre-exercise to *M* = 18.5, SE = 2.3 post-exercise, *p* < 0.001); Significant improvement in function according to CIPN-R-ODS (*M* = 79.9, SE = 2.7 pre-exercise to *M* = 83.2, SE = 2.4 post-exercise, *p* = 0.04).
Tonezzer et al. (2017)[73]	*Purpose*: Evaluate the effects of TENS on CIPN symptoms. Intervention group: 60 min of TENS daily for 45 days. Control: As with intervention with a sham machine	Visual Analogue Scale (VAS) assessing pain and other neuropathy-related symptoms. Chemotherapy-Induced Neurotoxicity Questionnaire (CINQ) to evaluate frequency of symptoms and interferences with daily activities	No significant findings on VAS or CINQ

*PN =* peripheral neuropathy, *M* = mean; *SD* = standard deviation; *TENS* = transcutaneous nervous stimulation; *CI* = confidence interval; *CIPN* = chemotherapy-induced peripheral neuropathy; *Mdn* = median; *SE* = standard error

**Table 3:** Pain outcomes: summary of measures and results

First author (year)	Purpose and intervention	Outcome measures	Main findings
Nathan et al. (2017)[63]	*Purpose*: Evaluate the effectiveness of community-based mindfulness-based stress reduction (MBSR) courses to improve physical and mental functioning among patients with PDPN whose medical treatment has been optimized. Intervention: Nine sessions of MBSR: eight weekly, 2.5-h sessions and one 6-h session on a weekend day midway through the course. Control: Usual activities, offered the opportunity to enroll in a MBSR course once the study was complete	Brief Pain Inventory (BPI) score	BPI Pain Severity mean score decreased in the MBSR group (*M* = − 1.59, 95% CIs [− 2.29 to − 0.90]) between baseline and week 12, compared to the control group (*M* = 0.33, 95% CIs [− 0.12 to 0.78], *p* < 0.001), indicating a reduction in pain in the intervention group.
Yoo et al. (2015)[61]	*Purpose*: Explore the effect of a supervised, moderate-intensity aerobic exercise training intervention on pain and pain interference in daily life, specifically in people with DPN. Intervention: 16 weeks of supervised aerobic exercise 3 times a week, of 30 to 50 min duration. Control: None	Various measures of pain and pain interference using the Brief Pain Inventory Short Form for Diabetic Peripheral Neuropathy (BPI-DPN) and a questionnaire devised by the authors.	Significant reductions in pain interference observed with walking on mean BPI-DPN score (*M* = 4.93, SD = 3.03 pre to *M* = 3.29, SD = 2.89 post, *p* = 0.016), normal work (*M* = 5.39, SD = 3.32 pre to *M* = 3.79, SD *=* 3.04 post, *p* = 0.032), relationships with others (*M* = 3.96, SD *=* 3.53 pre to *M* = 1.29, SD = 1.27 post, *p* = 0.006), sleep (*M* = 5.11, SD = 3.04 pre to *M* = 3.50, SD = 3.03 post, *p* = 0.02), and overall pain interference (*M* = 4.65, SD = 2.70 pre to *M* = 2.97, SD = 2.22 post, *p* = 0.013).
Kluding et al. (2012)[64]	*Purpose*: Examine the feasibility of a supervised, moderately intense aerobic and resistance exercise program in people with diagnosed diabetic PN. Intervention: 10-week exercise program with both aerobic and strengthening elements, 3 to 4 times per week. Control: None	Michigan Neuropathy Screening Instrument (MNSI) symptom questionnaire	Worst pain MNSI mean score decreased (*M* = 62.4, SD = 26.7 pre-intervention to *M* = 44.3, SD = 35.1 post-intervention, *p* = 0.05).
Forst et al. (2004)[70]	*Purpose*: Investigate the efficacy and safety of the Salutaris (TENS) device in patients suffering from symptomatic diabetic neuropathy. Intervention: TENS therapy over 12 weeks, self-administered for at least 30 min per day. Control: Electrically inactive (sham) device	Various pain qualities were measured by the Neuropathy Total Symptom Score-6 (NTSS-6), i.e., lancinating, burning, and aching pain, numbness, prickling sensation, and allodynia; pain intensity measured using a Visual Analogue Scale (VAS).	Active TENS treatment resulted in a significant improvement in mean NTSS-6 score after 6 weeks (− 42%) and after 12 weeks (− 32%) of treatment (baseline *M* = 10.0, SD = 3.3, 6 weeks *M* = 5.8, SD = 5.0 (*p* < 0.05), 12 weeks *M* = 6.8, SD = 3.9, *p* = 0.05), in comparison to the control group (baseline *M* = 7.6, SD = 3.1; 6 weeks *M* = 8.1, SD = 5.1, (n.s.), 12 weeks *M* = 6.5, SD = 6.1 [n.s.]). Improvement in mean sub-NTSS scores within the intervention group, including numbness (*M* = 2.19, SD = 1.05 to *M* = 1.55, SD = 1.26; *p* < 0.05); lancinating pain (*M* = 1.58, SD = 1.09 to *M* = 0.58, SD = 0.86; *p* < 0.05) and allodynia (*M* = 1.44, SD = 1.59 to *M* = 0.53, SD = 1.03; *p* < 0.05). The intervention group also reported a significant improvement in intervention group mean VAS score after 6 weeks of TENS therapy (*M* = 19.8, SD = 5.0 to *M* = 14.4, SD = 9.6; *p* < 0.05), while no change was observed in the control group.
Kumar and Marshall (1997)[69]	*Purpose*: Evaluate the efficacy of transcutaneous electrotherapy for chronic painful peripheral neuropathy in patients with type 2 diabetes. Intervention: TENS therapy over 4 weeks, self-administered for 30 min per day. Control: Electrically inactive (sham) device	Grading of pain intensity on a 0–5-point scale; perception of pain using a VAS	Significant reduction in pain grading for those in the intervention group, with the intervention group mean score declining from *M* = 3.17, SD = 0.12 to *M* = 1.44, SD = 0.25 (*p* < 0.01). Significant reduction also reported in the control group, with group mean score declining from *M* = 2.92, SD = 0.13 to *M* = 2.38, SD = 0.26 (*p* = 0.04). Intervention group reported greater reductions in pain through the VAS (*M* = 52, SD = 7) compared with the control group (*M* = 27, SD = 10, *p* < 0.05).
Serry et al. (2015)[71]	*Purpose*: To investigate the efficacy of TENS versus aerobic exercise and to compare them with regular pharmacological therapy in patients with diabetic PN. Group A: Received TENS therapy for 30 min 3 times per week for 8 weeks in addition to regular pharmacological therapy for PN. Group B: Engaged in aerobic exercise for 30 min 3 times for week for 8 weeks in addition to regular pharmacological therapy for PN. Group C: Received only regular pharmacological therapy for PN and oral hypoglycemic drugs or insulin.	Pain intensity evaluated using a VAS.	Compared to pharmacologic management, both the TENS and exercise groups had significant reductions in mean VAS pain score (TENS group 41.6% reduction, exercise group 16.7% reduction, *p* < 0.05). Group C (pharmacological management) showed no significant reductions in pain intensity.
Reichstein et al. (2005)[74]	*Purpose*: To compare the effects of high-frequency muscle stimulation (HF) with TENS therapy in patients with diabetic PN. HF group received HF for 30 min for 3 consecutive days. TENS group received TENS therapy for 30 min for 3 consecutive days.	1–10 point Numerical Rating Scale (NRS) for pain, numbness, burning, parasthesias; or dysesthesia taken at baseline, after all treatment days and 2 days post-intervention.	Reductions in NRS pre and post both interventions; 7 out of 21 patients (33%) in the TENS group and 16 out of 20 patients (80%) in the HF group reported improvement in pain symptoms (*p* < 0.05). HF more effective than TENS therapy in patients with nonpainful DPN (HF—100%, 7 out of 7; TENS—44%, 4 out of 9; *p* < 0.05), and in patients with painful DPN (HF—69%, 9 out of 13; TENS—25%, 3 out of 12; *p* < 0.05).
Gewandter et al. (2018)[72]	*Purpose*: To inform a future randomized phase 2 study and determine if TENS has the potential to improve CIPN. Intervention: Wireless TENS therapy for at least 1 h twice per day for a 6-week period. Control: None	Short Form McGill Pain Questionnaire-2 (SF-MPQ-2); daily diary containing NRS from 0 to 10 for pain, numbness, tingling, and cramping	NRS median scores indicated reduced pain symptoms post-TENS: pain reduced from a baseline median of 6 (IQR 1.8–6.6) to Mdn 3.7 (IQR 0.42–5.5, *p* < 0.001), tingling reduced from a baseline median of 5.7 (IQR 4.1–7.1) to Mdn 4 (IQR 1.8–5.1, *p* = 0.002) post-intervention, numbness from baseline median of 5.5 (IQR 4.7–6.6) to Mdn 4.4 (IQR 3.0–5.2, *p* < 0.001) post-intervention, and cramping from baseline median 3.0 (IQR 0.3–5.8) to Mdn 1.4 (IQR 0.0–3.1, *p* < 0.001) post-intervention.
Tonezzer et al. (2017)[73]	*Purpose*: To evaluate the effects of TENS on CIPN symptoms. Intervention group: 60 min of TENS daily for 45 days. Control: As with intervention with a sham machine	VAS assessing pain and other neuropathy-related symptoms	No significant findings on VAS
Teixeira (2010)[62]	*Purpose*: The purpose of this pilot study was to evaluate the effectiveness of mindfulness meditation on QoL among adults living with symptomatic diabetic PN. Intervention: The treatment group received instruction in mindfulness meditation and were instructed to listen to guided CD 5 days per week over a 4-week period. Control: Nutritional advice and asked to maintain a food diary for 4 weeks	Pain level using Neuropathic Pain Scale (NPS)	No statistically significant differences between the groups’ post-intervention scores on the NPS

*PDPN* = painful diabetic peripheral neuropathy; *M* = mean; *CI* = confidence interval; *DPN* = diabetic peripheral neuropathy; *SD* = standard deviation; *PN* = peripheral neuropathy; *TENS* = transcutaneous electrical nervous stimulation; *n.s.* = not significant; *CIPN* = chemotherapy-induced peripheral neuropathy; *IQR* = interquartile range; *Mdn* = median

**Table 4:** Quality of life outcomes: summary of measures and results

First author (year)	Purpose and intervention	Outcome measures	Main findings
Clark et al. (2012)[60]	*Purpose*: Determine the feasibility of using 3 complementary interventions in relieving the physical and emotional symptoms associated with CIPN while increasing capacity for mindfulness or self-focused attention. Intervention: A. Reiki intervention (*n* = 7)—up to 5 sessions over a 6-week period B. Yoga intervention (*n* = 7)—weekly session over 6 weeks C. Meditation intervention (*n* = 5)—weekly session over 6 weeks Control: One hour holistic education weekly for 6 weeks	Quality of life and PN using the Functional Assessment of Cancer Therapies—Gynecologic Group—Neurotoxicity Scale (FACT/GOG-NTx)	No significant difference between intervention groups on FACT/GOG-NTx. Subjects in the control group demonstrated significantly higher levels of neurotoxicity related QoL according to mean FACT/GOG-NTx score (*M* = 31.14, SD = 8.47 pre-test, *M =* 27.86, SD *=* 9.82 post-test, *p* = 0.037).
Park and Park (2015)[59]	*Purpose*: Analyze the effects of foot bathing and massage in patients with CIPN. Intervention group: One 30-min foot bath (temp 40 °C) session every other day, totaling 8 times over 2 weeks. Control: 30 min general massage sessions every other day, for a total of 8 times over 2 weeks	Quality of life (QoL) assessed using Functional Assessment of Cancer Therapy-General (FACT-G) and Functional Assessment of Cancer Therapy/Gynecologic Oncology Group/Neurotoxicity (FACT/GOG-NTx)	QoL (mean FACT-G score) increased in the foot bath group post-intervention (*M* = 62.75, SD = 11.29 pre-intervention vs *M* = 65.33, SD = 12.96 post-intervention, *p* = 0.028). Significant decrease in QoL in the foot massage group post-intervention (*M* = 59.63, SD = 12.47 pre-intervention vs *M* = 53.33, SD = 11.09 post-intervention, *p* = 0.042). QOL related to the symptoms (mean FACT-NTx score) in the foot bath group increased over time (*M* = 26.79, SD = 4.81 pre-intervention vs *M* = 31.13, SD = 5.57 post-intervention, *p* < 0.001), in comparison to QoL in the foot massage group which decreased over time (*M* = 29.42, SD = 7.82 pre-intervention vs *M* = 26.38, SD = 7.75 post-intervention, *p* < 0.001).
Dixit et al. (2014)[66]	*Purpose*: Evaluate the effect of 8-week moderate-intensity aerobic (heart-rate reserve 40–60%) exercise on neuropathy quality of life in people with diabetic PN. Intervention: Exercise training in the range of 40–60% of heart-rate reserve (HRR) within a rating of perceived exertion (RPE) (scale ranging from 6 to 20). Delivered 5–6 days of the week for 8 weeks, accumulating a minimum of 150 min/week to a maximum of 360 min/week. Control: Standard medical care, education for foot care and diet	Neuropathy quality of life (NQOL) score	Intervention group reported a reduction in mean NQOL score (*M* = 32.85, SD = 1.32, 95% CIs [33.28–32.42], decreased to *M* = 24.41, SD = 1.12, 95% CIs [24.82–24.00]), while the control group reported an increase in mean NQOL score (*M* = 33.55, SD = 1.37, 95% CIs [33.95–33.15], increased to *M* = 34.16, SD = 1.37, 95% CIs [34.61–33.71], *p* < 0.001)
Nathan et al., (2017)[63]	*Purpose*: Evaluate the effectiveness of community-based mindfulness-based stress reduction (MBSR) courses to improve physical and mental functioning among patients with PDPN whose medical treatment has been optimized. Intervention: Nine sessions of MBSR: eight weekly, 2.5-h sessions and one 6-h session on a weekend day midway through the course. Control: Usual activities, offered the opportunity to enroll in a MBSR course once the study was complete	Patient Global Impression of Change (PGIC) for QoL; Short Form-12 Health Survey version 2 (SF-12); Neuropathy-Specific Quality of Life Questionnaire (NQoL)	14 of 30 in the MBSR group (46.7%) compared to 2 of 32 in the control group (6.2%) reported improvements in mean PGIC score at the 12-week follow-up (adjusted OR 18.8, 95% CIs [2.3–151.5], *p* = 0.007), indicating that they perceived an increase in general well-being. The MBSR group mean NQoL score indicated higher quality of life related to pain between baseline and week 12 (*M* = − 1.39, 95% CIs [− 2.16 to − 0.61]), compared to the control group (*M* = 0.90, 95% CIs [− 0.53 to 2.33], *p* = 0.006). All SF-12 subscale mean scores indicated improvement in reported symptoms for the MBSR group, with the exception of vitality and role emotion (*p* < 0.05).
McCrary et al. (2019)[68]	*Purpose*: Evaluate the impact of a multimodal exercise intervention on CIPN symptoms, functional deficits, and neurophysiologic parameters. Intervention: 8-week exercise intervention with resistance, balance, and cardio elements, 3 times per week. Control: 8-week pre-intervention control period	Quality of Life (QoL) (SF-36)	Significant improvement in QoL (SF-36) (*M* = 60.5, SE = 3.7 pre-exercise to *M* = 69.1, SE = 3.6 post-exercise, *p* = 0.003)
Ruhland and Shields (1997)[65]	*Purpose*: Examine the effects of a home exercise program on PN impairment and QoL. Intervention: Exercise with Thera-Bands and cycling or walking for 10 to 20 min, over 6 weeks. Control: Maintain current levels of activity	Medical Outcomes Study (MOS) 36-Item Short-Form Health Survey (SF-36)	Significant improvement within the role limitation (physical) dimension of the SF-36, with mean scores increasing from *M* = 28.6 pre-test to *M* = 53.6 post-test (*p* = 0.007) for the exercise group compared to *M* = 55.4 pre-test to *M* = 62.5 (n.s.) for the control group.
Ahn and Song (2012)[67]	*Purpose*: Determine the effects of tai chi exercise on glucose control, neuropathy scores, balance, and quality of life in patients with diabetic PN. Intervention: One hour of tai chi per session, twice a week for 12 weeks. Control: Usual care	Subjective: Korean version of the SF-36v2 (36-Item Short Form Health Survey version 2)	Significant improvement in mean SF-36v2 score for several subsets including physical function (*p* = 0.028), bodily pain (*p* = 0.009), physical role limitation (*p* = 0.006), emotional role limitation (*p* = 0.002), and social functioning (*p* = 0.001)
Teixeira (2010)[62]	*Purpose*: Evaluate the effectiveness of mindfulness meditation on quality of life among adults living with symptomatic diabetic PN. Intervention: Treatment group received instruction in mindfulness meditation and were instructed to listen to guided CD 5 days per week over a 4-week period. Control: Nutritional advice and asked to maintain a food diary for 4 weeks	Quality of life using NQoL	No significant differences for adjusted overall QoL; symptom-related QoL; emotion-related QoL; sensory-motor related QoL; pain QoL

*CIPN* = chemotherapy-induced peripheral neuropathy; *M* = mean; *SD* = standard deviation; *PN* = peripheral neuropathy; *CI* = confidence interval; *PDPN* = painful diabetic peripheral neuropathy; *OR* = odds ratio; *SE* = standard error; *n.s.* = not significant

**Table 5:** Other outcomes: summary of measures and results

First author (year)	Purpose and intervention	Outcome measures	Main findings
Nathan et al. (2017)[63]	*Purpose*: Evaluate the effectiveness of community-based mindfulness-based stress reduction (MBSR) courses to improve physical and mental functioning among patients with PDPN whose medical treatment has been optimized. Intervention: Nine sessions of MBSR: eight weekly, 2.5-h sessions and one 6-h session on a weekend day midway through the course. Control: Usual activities, offered the opportunity to enroll in a MBSR course once the study was complete.	Glycosylated hemoglobin (HbA1c) measurement; Patient Health Questionnaire-9 (PHQ-9) for depression; Profile of Mood States-2A (POMS-2A) for total mood disturbance; Perceived Stress Scale (PSS); Pain Catastrophizing Scale (PCS)	Mean PCS score decreased in the MBSR group (− 10.67, 95% CIs [− 14.38 to − 6.95]), between baseline and week 12, compared to the control group (1.69, 95% CIs [− 1.47 to 4.85], *p* < 0.001). Mean PHQ-9 score significantly reduced in the MBSR group (*M* = − 4.75, 95% CIs [− 6.55 to − 2.96], *p* < 0.001), between baseline and week 12, compared to the control group (*M* = 0.06, 95% CIs [− 1.66 to 1.53], *p* < 0.001). The MBSR group mean PSS reduced between baseline and week 12 (*M* = − 4.64, 95% CIs [− 7.89 to − 1.38], *p* = 0.001), compared to the control group (*M* = 1.75, 95% CIs [− 0.14 to 3.64], *p* = 0.001).
Clark et al. (2012)[60]	*Purpose*: Determine the feasibility of using 3 complementary interventions in relieving the physical and emotional symptoms associated with CIPN while increasing the capacity for mindfulness or self-focused attention. Intervention: D. Reiki intervention (*n* = 7)—up to 5 sessions over a 6-week period E. Yoga intervention (*n* = 7)—weekly session over 6 weeks F. Meditation intervention (*n* = 5)—weekly session over 6 weeks Control: 1 h holistic education weekly for 6 weeks	Psychological distress using the Brief Symptom Inventory (BSI); Mindfulness using the Mindful Awareness Attention Scale (MAAS).	No significant difference between groups on BSI and MAAS
Ahn and Song (2012)[67]	*Purpose*: Determine the effects of tai chi exercise on glucose control, neuropathy scores, balance, and quality of life in patients with diabetic PN. Intervention: Standardized tai chi for diabetes of 1 h of tai chi per session, twice a week for 12 weeks. Control: Usual care	Fasting blood sugar (FBS); HbA1c; Semmes–Weinstein 10-g monofilament examination scores (SWME); single leg stance for balance.	Mean FBS reduced in the intervention group (*M* = 137.85 mg/dL, SD = 45.19 to 125.5 mg/dL, SD = 45.57, *p* = 0.036) compared to the control group (*M* = 143.47 mg/dL, SD = 47.45 to 155.31 mg/dL, SD = 44.88, *p* = 0.036). A significant difference was seen in HbA1c levels post-intervention in the tai chi group (*M* = 7.20, SD = 1.32, *p* = 0.004) compared to the control group (*M* = 8.32, SD = 1.76, *p* = 0.004). Differences in pre- and post-test balance mean scores indicated significant improvement in balance for the tai chi group (*M* = − 7.65, SD = 16.78, *p* = 0.044), compared to the control group (*M* = 1.44, SD = 9.97, *p* = 0.044). The SWME identified no significant changes in peripheral sensory function pre- and post-test.
Kluding et al. (2012)[64]	*Purpose*: Examine the feasibility of a supervised, moderately intense aerobic and resistance exercise program in people with diagnosed diabetic PN. Intervention: 10-week exercise program with both aerobic and strengthening elements, 3 to 4 times per week. Control: None	BMI; Resting heart rate (RHR) Glycosylated hemoglobin (HbA1c); Michigan Neuropathy Screening Instrument (MNSI) physical exam score; Nerve conduction studies (NCS); Quantitative Sensory Testing (QST); intraepidermal nerve fiber density (IENF)	Significant reduction in HbA1c (*M* = 7.8, SD = 1.0 pre-intervention to *M* = 7.28, SD = 0.83 post-intervention, *p* = 0.031). Significant increase in IENF branching at the proximal biopsy site (*M* = 0.16, SD = 0.15 pre-intervention to *M* = 0.27, SD = 0.19 post-intervention, *p* = 0.008). Significant reduction in RHR (*M* = 77.3, SD = 8.2 pre-intervention to *M* = 72, SD = 9.6 post-intervention, *p* = 0.036). No significant difference in NCS or QST
Ruhland and Shields (1997)[65]	*Purpose*: Examine the effects of a home exercise program on PN impairment and quality of life. Intervention: Exercise with Thera-Bands and cycling or walking for 10 to 20 min, over 6 weeks. Control: Maintain current levels of activity	Average Muscle Score (AMS); handgrip force; forced vital capacity (FVC); timed 9.1 m walk	Significant improvement in mean AMS in exercise group (pre-test *M* = 8.8, post-test *M* = 9.2, *p* = 0.002); significant improvement in handgrip force (pre-test *M* = 28.6, post-test *M* = 30.8, *p* = 0.033)
Yoo et al. (2015)[61]	*Purpose*: Explore the effect of a supervised, moderate-intensity aerobic exercise training intervention on pain and pain interference in daily life, specifically in people with DPN. Intervention: 16 weeks of supervised aerobic exercise 3 times a week, of 30 to 50 min duration. Control: None	Objective: body mass index (BMI); aerobic fitness (VO2max); blood pressure; glycemic control (hemoglobin A1c)	No significant changes were found for BMI, blood pressure, or glycemic control. Significant improvement in mean maximum oxygen uptake (VO2max) (mL/kg/min) (*M* = 16.02, SD = 3.84 pre to *M* = 17.18, SD = 4.19 post, *p* = 0.028).
Gewandter et al. (2018)[72]	*Purpose*: To inform a future randomized phase 2 study and determine if TENS has the potential to improve CIPN. Intervention: Wireless TENS therapy for at least 1 h twice per day for a 6-week period. Control: None	Objective: Utah Early Neuropathy Score (UENS); forced choice monofilament test	No significant improvements with UENS; monofilament test reported sensation threshold improved in 10 of 16 (63%; 95% CI [35–85%], *p* < 0.0001) participants who completed the test.
Serry et al. (2015)[71]	*Purpose*: To investigate the efficacy of TENS versus aerobic exercise, and to compare them with regular pharmacological therapy in patients with diabetic PN. Group A: Received TENS therapy for 30 min 3 times per week for 8 weeks in addition to regular pharmacological therapy for PN. Group B: Engaged in aerobic exercise for 30 min 3 times for week for 8 weeks in addition to regular pharmacological therapy for PN. Group C: Received only regular pharmacological therapy for PN and oral hypoglycemic drugs or insulin.	Nerve conduction studies (NCS) to measure medial plantar sensory nerve conduction velocity (SCV)	No significant differences in SCV between pre- and post-test measurements for any of the groups, or between the groups
McCrary et al. (2019)[68]	*Purpose*: Evaluate the impact of a multimodal exercise intervention on CIPN symptoms, functional deficits and neurophysiologic parameters. Intervention: 8-week exercise intervention with resistance, balance, and cardio elements, 3 times per week. Control: 8 week pre-intervention control period	Objective: Total Neuropathy Score Clinical version (TNSc); mobility (6 min timed walk); standing balance (Swaymeter); lower limb strength and dynamic balance (5 times sit to stand test)	Significant reduction in TNSc symptom score (*M* = 7.0, SE = 0.7 pre-exercise to *M* = 5.3, SE = 0.5 post-exercise, *p* = 0.001); significant increase in distance in 6 min walk test (m) (*M* = 452.1, SE = 17.4 pre-exercise to *M* = 469.9, SE = 20.9 post-exercise, *p* = 0.02); significant reduction in 5 times sit to stand time (s) (*M* = 13.1, SE = 0.8 pre-exercise to *M* = 11.8, SE = 0.6 post-exercise, *p* = 0.03); significant reduction in postural sway (mm) on a stable surface eyes open (*M* = 140.9, SE = 23.6 pre-exercise to *M* = 104.2, SE = 13.6 post-exercise, *p* = 0.006)

*PDPN* = painful diabetic peripheral neuropathy; *CI* = confidence interval; *M* = mean; *mg* = milligram; *dL* = deciliter; *SD* = standard deviation; *PN* = peripheral neuropathy; *kg* = kilogram; *min* = minute; *DPN* = diabetic peripheral neuropathy; *TENS* = transcutaneous electrical nervous stimulation; *CIPN* = chemotherapy-induced peripheral neuropathy; *SE* = standard error

### Study quality assessment and data synthesis

Study designs and approaches were evaluated for risk of bias by two reviewers using domains of the Cochrane Risk of Bias Tool (Table 6). Study outcomes were evaluated through a narrative synthesis instead of a meta-analysis due to the heterogeneous etiologies for PN and the variety of self-initiated self-management strategies and outcomes evaluated.

**Table 6:**
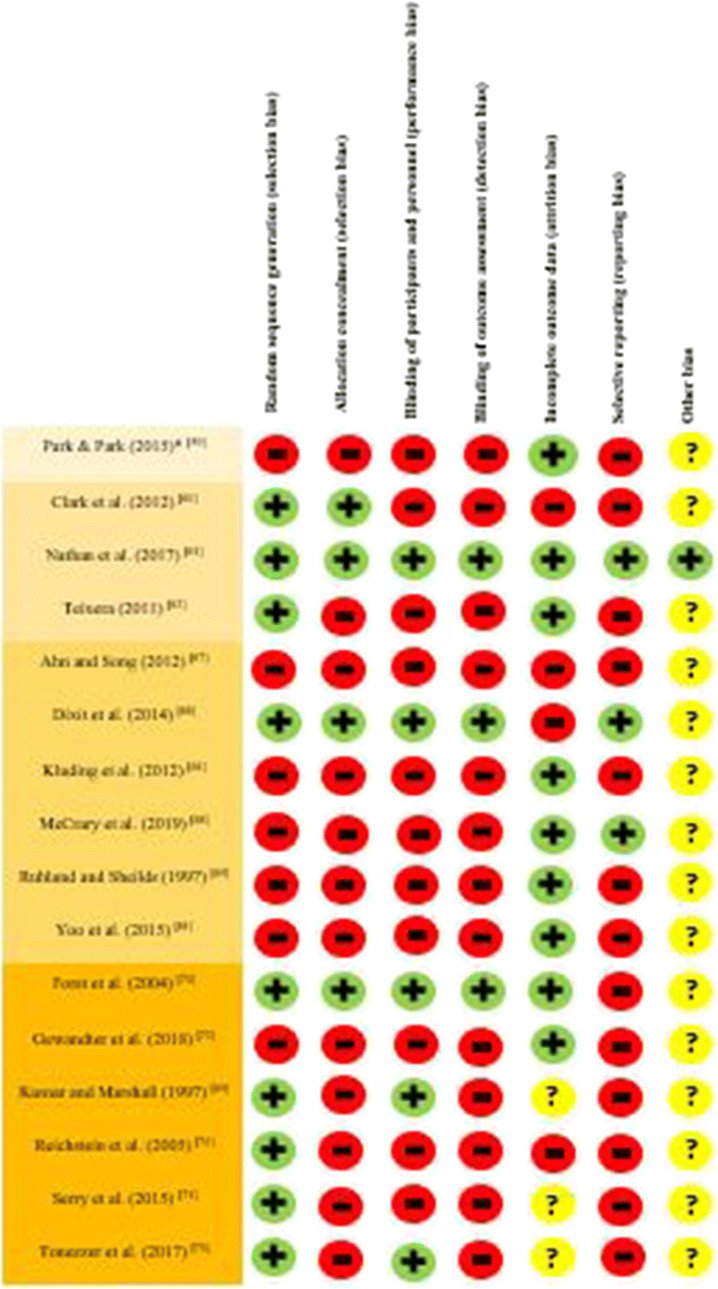
Quality assessment of the included studies

*Studies have been grouped according to intervention type

## Results

### Literature search

**Fig. 1:**
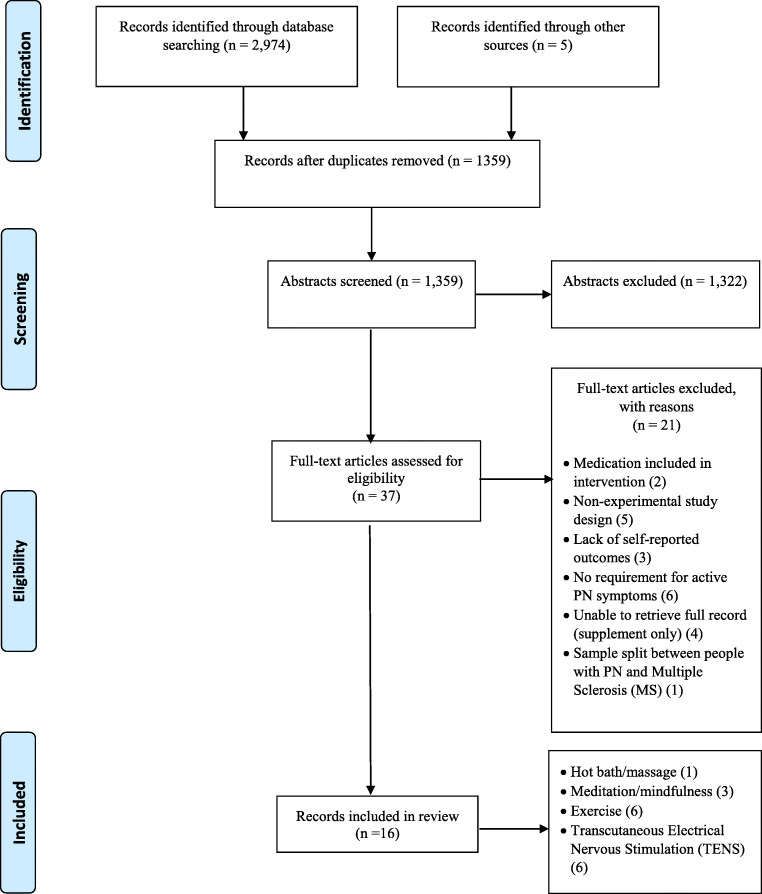
Study selection process

An initial database search identified 2979 articles (with reference screening identifying an additional 5 papers), of which 1620 were duplicates. Following the review of 1322 abstracts, 37 full text articles were assessed for eligibility (Fig. 1). Sixteen studies were deemed eligible for inclusion in the review (Tables 1, 2, 3, 4, and 5). The primary reasons for exclusion were medication included in the intervention (*n* = 2); nonexperimental study design (*n* = 5); lack of self-reported outcomes (*n* = 3); patients not required to have symptoms of PN (*n* = 6); patients split between those with PN and multiple sclerosis (MS) (*n* = 1); and articles available only as abstracts, with insufficient information available in the record to determine whether the study met the stated objectives (*n* = 4).

### Description of studies

Studies were identified that assessed the following self-initiated strategies: massage and hot baths (within a single study [59]), meditation [60, 62, 63], exercise [61, 64–68], and TENS [69–74]. The TENS interventions were included because in some settings this treatment is initiated by patients themselves (i.e., TENS machines are available for purchase over the counter in the United States [US], some European countries, and Australia). No cold/ice pack or magnet studies were identified that fit the criteria for inclusion in this review. Seven of the 16 studies were conducted in the US [60–62, 64, 65, 69, 72], with the remaining nine conducted in South Korea [59, 67], Germany [70, 74], India [66], Canada [63], Egypt [71], Australia [68], and Brazil [73]. Nine of the 16 studies involved randomized controlled trial designs [60, 62, 63, 66, 69–71, 73, 74]. Two of the studies involved nonrandomized allocation to intervention and control groups [59, 65]. The remaining five studies employed a pre- and post-test design [61, 64, 67, 68, 72].

The 16 studies included a total of 549 patients, with sample sizes ranging from 14 [61] to 66 [66]. PN arose from a variety of disease and treatment-related etiologies, including CIPN [59, 60, 68, 72, 73], DPN [61–64, 66, 67, 69–71, 74], and CIDN [65]. The mean age of the patients ranged from a low of 49.5 years [73] to a high of 74.6 years [62]. Nine of the 16 studies had more than 50% female patients, with one study reporting that 88.5% of the patients were female [60]. Studies varied considerably in their reported attrition rates, from a low of 9% [63, 65] to a high of 37% [64]. Four studies did not report attrition rates [69, 71, 73, 74]. Table 6 summarizes the potential bias across the identified studies.

### Summary of findings

#### Self-reported PN-associated symptoms and impairment

Seven of the 16 studies measured PN symptoms pre- and post-intervention, using seven different instruments [64, 66–68, 72–74]. Detailed results for PN-associated symptom and impairment measures and outcomes are included in Table 2. Ahn and Song found a moderately significant difference in mean Total Symptom Score (TSS) between the exercise (tai chi) and control groups, indicating an improvement in PN-related impairment post-exercise [67]. In another study that utilized the TSS to compare the effectiveness of TENS and high-frequency external muscle stimulation (HF) [74], reductions in mean TSS score were found for both the high-frequency muscle stimulation group and the TENS group, with the high-frequency muscle stimulation group reporting larger symptom reductions than the TENS group.

Dixit et al. found significant improvements in mean Michigan Diabetic Neuropathy Scores (MDNS) across sensory, motor, and reflex subscales between baseline and the 8th week for the exercise group compared to the control group [66]. Kluding et al. similarly reported significant improvements in neuropathy impairment scores following a single arm exercise intervention, with overall mean Michigan Neuropathy Screening Instrument (MNSI) symptom scores decreasing significantly post-exercise intervention [64]. In a single arm study that utilized the European Organization for Research and Treatment of Cancer-CIPN20 (EORTC-CIPN20) to identify self-reported changes in CIPN symptoms [72], median baseline scores reduced following a TENS intervention. In another study that utilized the EORTC-CIPN20 to identify changes in symptom burden pre and post an exercise intervention [68], mean scores decreased significantly [68]. In the same study, the CIPN Rasch Built Overall Disability Score (CIPN-R-ODS) was used to identify self-reported changes in functional abilities pre and post the exercise intervention. Mean scores on the CIPN-R-ODS demonstrated a statistically significant increase [68]. In another study that used the Chemotherapy-Induced Neurotoxicity Questionnaire (CINQ) to evaluate the frequency and impact of acute and chronic CIPN symptoms on ability to conduct daily activities pre- and post-TENS intervention, no significant changes in neuropathic symptoms were reported [73].

#### Pain symptoms associated with PN

Ten of the 16 studies assessed pain using 11 different instruments [61–63, 69–74]. Detailed results for PN-associated pain measures and outcomes are included in Table 3. Two of the studies used the Brief Pain Inventory (BPI) [63] and the Brief Pain Inventory Short Form for Diabetic Peripheral Neuropathy (BPI-DPN) [61]. Nathan et al. reported significant decreases in mean BPI Pain Severity scores in the mindfulness group between baseline and week 12, compared to the control group over the same time period [63]. In a single arm exercise study, Yoo et al. reported significant reductions in mean BPI-DPN interference scores for walking, normal work, relationships with others, sleep, and overall pain interference [61].

All of the TENS studies utilized a Visual Analogue Scale (VAS) or Numerical Rating Scale (NRS) to measure pain pre- and post-intervention [69–74]. Three of the TENS studies reported mean reductions in the intervention groups’ VAS scores between pre- and post-intervention [69–71]. Significant differences in pre-/post-intervention mean pain for the TENS and control groups were reported, with the intervention group reporting greater reductions. Additionally, nine participants in the control group who subsequently received the intervention reported reductions in mean pain scores following the intervention [69].

Reichstein et al. reported reductions in mean NRS scores following both high-frequency muscle stimulation and TENS interventions, with high-frequency muscle stimulation (HF) reportedly more effective than TENS therapy in patients with both painful and nonpainful DPN [74]. Tonezzer et al. reported no significant changes in the VAS score post-TENS intervention between or within the intervention and control groups [73]. The single arm study of Gewandter et al. used daily patient diaries including a NRS that ranged from 0 to 10 to assess changes to self-reported pain associated with CIPN following a TENS intervention [72]. Significant reductions in median scores from baseline to post-TENS were found across self-reported pain and other symptoms, including tingling, numbness, and cramping [72]. Gewandter et al. also reported decreases in self-reported pain using the Short Form McGill Pain Questionnaire-2 (SF-MPQ-2) [72].

Forst et al. used the New Total Symptom Score (NTSS-6) to measure a number of pain qualities (i.e., lancinating, burning, and aching pain, numbness, prickling sensation, and allodynia), identifying reductions in self-reported numbness, lancinating pain, and allodynia following the TENS intervention [70]. While a number of similar pain characteristics associated with DPN were assessed in a single arm study of exercise [61], no reportable pattern of changes were identified [61]. Another single arm exercise study identified that worst pain MNSI mean score decreased post-exercise [64]. While Teixeira assessed participants’ pain pre and post a meditation intervention using the Neuropathic Pain Scale (NPS), no significant changes were reported [62].

#### Quality of life

Eight of the 16 studies (massage/foot bath, meditation, and exercise) assessed quality of life using seven different measures [59, 60, 62, 63, 65–68]. Detailed results for PN-associated quality of life measures and outcomes are included in Table 4. Using the Functional Assessment of Cancer Therapies—Gynecologic Oncology Group—Neurotoxicity Scale (FACT/GOG-NTx), the meditation intervention of Clark et al. reported no significant changes in mean quality of life score within or between any of the four groups; however, patients in the control group reported significantly lower levels of symptom-related quality of life pre-/post-test [60]. In another study that used the FACT/GOG-NTx [59], mean reported symptom-related quality of life improved over time in the foot bath group in comparison to mean reported symptom-related quality of life in the foot massage group, which decreased over time [59]. In terms of overall quality of life, in the foot bath group, mean reported scores increased over time, indicating an increase in quality of life, while in the foot massage group, mean scores decreased over time [59].

Three studies assessed quality of life changes using the Neuropathy-Specific Quality of Life Questionnaire (NeuroQoL) [62, 63, 66]. Dixit et al. reported significant improvements for the exercise intervention group across various NeuroQoL dimensions, including pain, reducing feeling/sensation, sensory-motor symptoms, restriction in activity of daily living, disruptions in social relationships, emotional distress, specific impact on quality of life, and overall quality of life [66]. While Nathan et al. did not report a total NeuroQoL mean score for either group or subtests scores at each assessment point, significant differences were reported across a number of subdimensions between the meditation and control groups, including reductions in pain severity, pain interference with daily activities, pain interference with emotional and physical roles, and in the effect of pain symptoms on mood [63]. Nathan et al. also assessed perceived quality of life related to general health and well-being using the Short Form-12 Health Survey version 2 (SF-12) and the Patient Global Impression of Change (PGIC) [63]. Patients in the mindfulness group reported improvements in symptoms across all SF-12 subscales in comparison with the control group, with the exception of vitality and role emotion [63].

McCrary et al. [68], Ruhland and Shields [65], and Ahn and Song [67] used the 36-Item Short Form Health Survey to measure a range of health dimensions associated with quality of life [65, 67]. McCrary et al. reported a significant improvement in mean quality of life scores for the exercise intervention instrument overall [68]. Ruhland and Shields reported significant improvement within the role limitation (physical) dimension of the instrument for the exercise group compared to the control group [65]. Ahn and Song reported significant differences between pre- and post-test mean scores for several subsets of the SF-36v2 (Korean version) following the 12-week tai chi intervention, including improved physical function, improved bodily pain, improved physical role limitation, improved emotional role limitation, and improved social functioning [67].

#### Other outcomes

This review focused on studies that included measures of PN-related pain and quality of life. However, a number of the included studies used other outcome measures including mood symptoms and more objective measures of PN. While not the primary focus of this review, the key results are summarized below. Detailed results for mood symptoms and objective measures and outcomes are included in Table 5.

In terms of mood, two studies measured a range of psychological variables [60, 63]. Nathan et al. reported significant differences in Pain Catastrophizing Scale (PCS) scores, with patients in the mindfulness meditation group reporting significant reductions between 1 and 12 weeks [63]. Using the Patient Health Questionnaire-9 (PHQ-9), patients in the mindfulness meditation group reported more significant reductions in depression scores compared to the control group [63]. Using the Perceived Stress Scale (PSS), patients in the mindfulness meditation group reported more significant reductions in perceived stress compared to the control group [63]. The meditation study of Clark et al. did not identify any significant differences in psychological distress levels between groups’ mean scores for the Brief Symptom Inventory (BSI) [60].

A number of studies included objective measures of PN to assess the effectiveness of the intervention. Ahn and Song’s tai chi exercise study used a range of objective measures including fasting blood sugar (FBS), HbA1c, balance, and peripheral sensory function [67]. Mean FBS reduced in the tai chi group compared to the control group, with a significant difference also seen in HbA1c levels post-intervention in the tai chi group [67]. Differences in pre- and post-test balance scores indicated significant improvement in balance for the tai chi group. However, the Semmes–Weinstein monofilament examination (SWME) identified no significant changes in peripheral sensory function pre- and post-test [67].

The single arm exercise intervention in the study by Kluding et al. resulted in significant reductions in HbA1c and resting heart rate (RHR) post-intervention [64]. No significant differences were found in nerve conduction studies undertaken pre- and post-test, although punch biopsies revealed significant changes in proximal intraepidermal nerve fiber (IENF) density and branching [64]. Dixit et al. reported no significant changes in body mass index (BMI), waist to hip ratio (WHR), FBS, post-prandial blood sugar (PPBS), or nerve conduction studies between the exercise and control groups [66].

Ruhland and Shields found significant improvements in the exercise group in mean average muscle score (AMS) compared to the control group. No significant changes were observed for left handgrip force, forced vital capacity (FVC), timed walk, BMI, blood pressure, or glycemic control [65]. Yoo et al. reported a significant improvement in mean maximum oxygen uptake (VO2max) (mL/kg/min) post single armed exercise intervention. However, no significant changes in BMI, HbA1c, or blood pressure were observed [61]. Gewandter et al. reported no significant improvement in the Utah Early Neuropathy Score (UENS) between the TENS and control groups. However, improved sensation threshold was found in 10 of the 16 patients who completed the test [72]. Serry et al. reported no changes in sensory nerve conduction velocity (SCV) scores within or between the TENS, exercise, or pharmacological intervention groups [71].

McCrary et al. used a number of objective measures pre- and post-exercise intervention [68]. A significant reduction in TNSc symptom score indicated an improvement in CIPN symptom severity, and a significant increase in distance in the 6-min walk test (meters) indicated an improvement in mobility post-intervention [68]. A significant reduction in five times sit to stand time (seconds) indicated an improvement in lower limb strength and balance and a significant reduction in postural sway (millimeters) suggested an improvement in balance on a range of dimensions [68]. NCS did not identify significant changes in sensory or motor neurophysiological functioning post-intervention [68].

### Risk of bias

As identified in Table 6, 15 of the 16 studies include in this review had a high or unclear risk of bias. Several of the studies did not involve randomized designs [59, 61, 64, 65, 67, 68, 72], and of those that were randomized, only five reported that blinding occurred [63, 66, 69, 70, 73]. Many of the studies had small sample sizes, ranging from 14 [61] to 66 [66]. The lack of a placebo intervention or control group was cited by several authors as a significant limitation [59, 61, 64], as was failure to document time spent engaged in the intervention [69, 72]), with low or no adherence rates reported in the meditation studies [60, 72–74]. Some of the studies had methodological issues that limited the quality of findings, such as failure to sufficiently power the study to produce statistically significant outcomes [60, 62, 65, 67] or the inclusion of too many intervention groups limiting the sample sizes for comparisons [60].

Attrition rates varied considerably across the studies, from a low of 9% [63, 65] to a high of 37% [64], with one study suggesting that those who dropped out were younger and reported less severe symptoms [67]. Some studies did not control for confounders such as medication use [59, 62, 65, 71–74] or comorbidities that may affect or increase PN symptoms [62, 64]. Some studies were of limited duration (4 weeks or less, including one study that spanned 3 days [74]) and some did not take repeated measurements to confirm the validity and reliability of results [59, 62, 69, 74]. The majority of the studies did not report results in a manner that allowed comparison across studies, with the exception of Dixit et al., Nathan et al., and McCrary et al. [63, 66, 68].

A range of patient and methodological factors make comparisons across disease and intervention groups challenging, even across studies where the same instrument was applied to measure reductions in self-reported pain. While five of the six TENS studies [69–72, 74] used a VAS or NRS to assess pain reduction, deficiencies in the quality and consistency of reported results preclude meaningful comparison across the studies. Similarly, the FACT/GOG-NTx was used to measure quality of life in CIPN patients in both the hot bath/massage study and one of the meditation studies identified. However, limitations including lack of randomization, failure to blind participants and research personnel, and incomplete reported outcome data require that positive findings are considered with caution [59, 60].

Although five of the six TENS studies measured pain with a VAS or NRS, it is difficult to compare the results across the studies because the TENS machine settings were all reported differently [69–72, 74]. Patients were able to manipulate machine settings across the studies, and indeed were encouraged to do so in several of the studies [69–74]. The studies in this review did not attempt to identify whether symptoms were alleviated at a proportionally higher level at greater intensities of TENS administration [69–74].

The small research base necessitated the inclusion of studies in this review with samples experiencing PN from a variety of etiologies. While the symptoms of CIPN, HIV-related PN, and DPN may be expressed similarly, there may be important differences in these populations that mean comparisons across heterogeneous groups are not ideal. These differences may include variations in comorbidities, age, and ethnicity, which could affect individuals’ responses to self-management strategies to alleviate PN symptoms [75–79]. For example, HIV patients with neuropathy are more likely to be male and younger than those with diabetic neuropathy or CIPN, as diabetes and cancer incidence increase with age, and PN disproportionately affects women [77, 79, 80].

Furthermore, most studies did not control for baseline PN severity in the study design or data analyses. Half of the studies did not specify a minimum grade or severity of PN as part of the inclusion criteria, and only two stratified results according to symptom severity [63, 66]. False-positive findings may be reported if a study contains a large proportion of unaccounted for cases with favorable PN baseline and subsequent scores. Alternatively, the effectiveness of some interventions for certain subgroups with PN may be not be identified if masked by average scores of the total sample.

This review did not seek to identify studies which included objective measures of changes in PN. Nevertheless, four of the five studies that assessed exercise in improving DPN symptoms reported improvements in objective health or physiological measures [61, 64, 67, 68]. Ahn and Song reported improvements in blood glucose levels, as did Kluding et al. who also identified significant improvements in IENF branching through biopsy [64, 67]. Although Yoo et al. did not report significant changes to BMI, blood pressure, or glycemic control, patients’ general fitness (as measured by maximal oxygen uptake [VO2max]) did improve [61]. Studies have proposed that impaired insulin signaling and consequent insulin resistance in both metabolic and neuronal tissues are common factors in type 2 diabetes and diabetic PN, which suggests that efforts to reduce the impact of diabetes may be effective in reducing DPN symptoms [81, 82]. Even in the absence of statistically significant changes to anthropomorphic measures, exercise may work to reduce the severity of DPN in ways that may differ from the mechanisms of action for PN arising from other etiologies [66]. Therefore, comparisons of the effectiveness of exercise interventions across groups with PN arising from different etiologies should be undertaken with caution.

Future studies that examine the effectiveness of self-initiated symptom management strategies should include larger sample sizes to increase the power of studies, thereby increasing the ability of researchers to identify existing effects. Homogeneous patient groups would allow for more focused examination of the impact of self-initiated interventions and self-reported reductions in PN symptoms. Future research should seek to identify factors that affect the likelihood that people will engage in self-initiated strategies. More rigorous planning is required to better monitor adherence to strategies and control for potential confounders, including prescribed medications. Baseline symptom severity should also be considered through inclusion/exclusion criteria, or through stratification of symptom severity results reporting.

### Limitations

This review has various limitations. The review’s limited focus on self-initiated self-management activities and restriction of articles only published in English resulted in a small research base and necessitated the inclusion of studies in this review with samples experiencing PN from a variety of etiologies. While the symptoms of CIPN, HIV-related PN, and DPN may be expressed similarly, there may be important differences in these populations that mean comparisons across heterogeneous groups are not ideal. These differences may include variations in comorbidities, age, and ethnicity, which could affect individuals’ responses to self-management strategies to alleviate PN symptoms [64–68]. For example, HIV patients with neuropathy are more likely to be male and younger than those with diabetic neuropathy or CIPN, as diabetes and cancer incidence increase with age, and PN disproportionately affects women [66, 68, 69]. This could affect the generalizability of results across the broader population of people experiencing PN symptoms. Excluding articles not published in English may also have resulted in a cultural bias toward self-initiated activities conducted in developed or Western societies which may limit the extent to which these activities could be recommended across populations.

## Discussion

This review sought to identify the effectiveness of self-initiated self-management strategies in alleviating self-reported PN symptoms. This review suggests that a range of self-initiated strategies, including massage, hot baths, TENS therapy, exercise, and meditation, may be effective in reducing self-reported PN symptoms and may improve aspects of quality of life for those living with PN. Fourteen of the 16 studies recorded some positive effects regarding outcomes measured, including pain, quality of life, and general health [59, 61, 63–73]. These positive findings highlight the potential benefits of these interventions for patients with PN, although the high risk of bias in nearly all studies included in this review means results should be considered with caution. Further research in this area is needed.

Notwithstanding the limitations of available research, a number of possible explanations could account for the positive findings identified in this review. For example, in the case of DPN, adoption of various behavior changes, including diet modification and exercise, can reduce the severity of diabetes itself [61, 81, 82], thereby reducing the adverse effects of this disease. Five of the studies included in this review sought to determine the efficacy of exercise in reducing the symptoms of DPN [61, 64, 66–68], with all reporting reductions in symptom scores and improvements in quality of life score. While similarities in the expression and experience of DPN and PN of other etiologies such as CIPN or HIV-related PN have been reported, responses to different interventions may vary between the groups in accordance with the mechanism of action of those interventions [76, 77, 80].

While a direct causal relationship between the original mechanism of injury and the means to reverse this damage has been suggested only for DPN, other strategies such as heat therapy, exercise, massage, and TENS may have benefits beyond localized symptom management [45, 64]. Heat, massage, and exercise improve blood flow to affected areas, which increases tissue perfusion and oxygenation and enhances the removal of inflammatory mediators that promote cellular dysfunction [45, 61, 64]. The reduction in pain symptoms associated with TENS and exercise may be explained by decreases in pro-inflammatory cytokines and the release of endogenous opioids and serotonin [71, 83, 84]. In addition, heat therapy, massage, and TENS may reduce symptoms through stimulation of larger nerve fibers which influences pain perception [44–46, 84].

Although 14 of the 16 studies included in this review reported positive outcomes associated with a number of self-initiated self-management strategies for PN, it is difficult to compare these results within and between interventions due to the variety of instruments used to measure PN. Within the same intervention category, there was typically some variation with respect to the etiology of PN as well as the instruments used to measure PN symptoms, pain, and quality of life. This inconsistency makes it very difficult to compare the effectiveness of any intervention even when studies evaluated interventions for PN that arose from the same etiology. Future research would benefit from consistency in the use of instruments to measure PN symptoms, pain, and quality of life.

This review highlighted other considerations when undertaking symptom management studies. For example, while not the primary focus for this review, several studies involved the use of objective outcome measures, such as nerve conduction, in addition to self-reported measures. Pre- and post-intervention changes were not observed using these objective measures, even where patients’ self-reported perception of symptoms did reflect improvements [64, 66, 67, 71]. With the exception of McCrary et al. [68], who suggested that self-reported improvements in CIPN symptom severity may be related to perceived improvements in quality of life or through adaptation to deficits over time, the studies in this review did not explore possible reasons for the differences in findings when both objective and subjective measures were used [68]. Nevertheless, these differences highlight the importance of including self-report measures in symptom management research, given the subjective and deeply personal nature of how symptoms from a range of chronic conditions are experienced [34, 85, 86]. Subjectively perceived changes in symptom experiences are worthy of investigation in their own right, as important changes in patient experiences may be overlooked if objective measures alone are used [87]. These findings provide further support for symptom management theories, such as the theory of symptom management [88], which highlight the importance of an individual’s perceptions of symptoms and the relationship between these perceptions and the use of self-management strategies.

## Conclusions

This review identified that studies that evaluated the effectiveness of heat therapy, exercise, TENS, and mindfulness/meditation interventions to reduce the symptoms of PN, or improve quality of life for those affected, report predominantly favorable results. However, the low quality of the studies included in this review means that recommendations should be made with caution. Although a few studies identified the strategies that people with PN engage in to manage their symptoms independently [22–25], the studies considered in this review suggest that the potential benefits from interventions such as heat therapy, meditation/mindfulness, exercise, or TENS therapy for people with PN in terms of symptom relief and improved quality of life warrant further investigation [37, 38, 89].

Clinicians have a role in encouraging patients to identify strategies to reduce PN-related symptoms and to determine the efficacy of interventions on a trial and error basis. While studies of self-initiated self-management interventions to date have not been of high quality, available findings suggest that some patients with PN symptoms report that heat therapy, exercise, mindfulness/meditation, or TENS was effective in reducing their symptoms. Just as the perception of pain and other symptoms associated with PN is experienced on a very individual basis, so is the perception of respite from symptoms. Therefore, patients may therefore benefit from experimenting with the application of these strategies [25] given that they are for the most part associated with minimal risk. Health care professionals can empower patients by directing them to resources that will allow them to make informed decisions about strategies that may work for them, while helping them to understand any risks and safety considerations required with individual strategies aimed at reducing PN symptoms [37, 90].
